# Ecotoxic effect of mycogenic silver nanoparticles in water and soil environment

**DOI:** 10.1038/s41598-025-95485-x

**Published:** 2025-03-28

**Authors:** Aleksandra Tończyk, Katarzyna Niedziałkowska, Katarzyna Lisowska

**Affiliations:** 1 Department of Industrial Microbiology and Biotechnology, Faculty of Biology and Environmental Protection, University of Lodz, 12/16 Banacha Street, Lodz, 90-237 Poland; 2 The BioMedChem Doctoral School of University of Lodz and Lodz Institutes of Polish Academy of Sciences, 21/23 Matejki Street, Lodz, 90-237 Poland

**Keywords:** Silver nanoparticles, Biosynthesis, Mycogenic, Ecotoxicity, Soil ecosystems, Water ecosystems, Nanobiotechnology, Environmental impact

## Abstract

Silver nanoparticles (AgNPs) are one of the most widely used nanomaterials due to their antimicrobial properties. Among the AgNPs synthesis methods, the biological route has become preferable because of its efficiency and eco-friendly character. Filamentous fungi can be successfully used in biosynthesis of AgNPs. The extensive application of AgNPs and their ever increasing production raise concerns about their environmental safety. AgNPs can be released during manufacturing processes or by leaching from AgNPs-supplemented products, and then enter the natural environment. Water and soil ecosystems are most exposed to the AgNPs presence. The present study aimed at evaluating the ecotoxicological potential of AgNPs derived from *Gloeophyllum striatum* fungus. The assessment was performed using organisms from water and soil ecosystems. Our results suggest that the presence of AgNPs can threaten the organisms inhabiting exposed ecosystems and the adverse effects of AgNPs differ depending on the organism species. Freshwater crustacean *Daphnia magna* was found to be the most sensitive among the tested species with EC_50_ values ranging 0.026–0.027 µg/mL after 48 h exposure. Crop plants were the least affected by the presence of AgNPs with EC_50_ values above tested AgNPs concentration range. Moreover, it was noted that ecotoxicological potential varied depending on the AgNPs synthesis scheme and these differences were the most visible in the case of *S. polyrhiza*.

## Introduction

Nanomaterials have gained broad utility due to their unique properties^1^. Among them, silver nanoparticles (AgNPs) seem to be the most recognizable, as they constitute 25% of all nanoparticles used in commercial products^2^. The antimicrobial potential of silver itself has been known since ancient times^3^ and such a widespread use of AgNPs is mostly due to this property. They are exploited in various areas such as medicine, biotechnology, personal care and cosmetics, textile or food industry^1,4^. Moreover, AgNPs exhibit some physicochemical qualities making them useful in technical fields, e.g. engineering, microelectronics or environmental remediation^1,3^.

The world annual production of AgNPs is expected to exceed 800 tons by the year 2025^5^. The extensive use of products containing AgNPs has led to the increased presence of these nanoparticles in the natural environment^6^. AgNPs can be accidentally released during the production process, distribution, use and disposal of the product. The most exposed ecosystems are water and terrestrial environments, where AgNPs are transferred mostly by wastewater and sewage sludge used in agriculture as fertilizer^2,3^. The presence of AgNPs in the environment raises concerns regarding their toxicity towards organisms that inhabit endangered ecosystems. Moreover, there is a risk of AgNPs entering parts of the food chain such as plants and animals, which can pose a serious threat to the human health. The knowledge of AgNPs toxicity mechanisms as well as short- and long-term exposure effects is scarce^1,2^. Therefore, research concerning the environmental impact of AgNPs is needed to enable designing efficient and safe antimicrobial agents.

AgNPs can be synthesized by three methods: chemical, physical and biological, also known as green synthesis. The last one is considered to be more environmentally friendly compared to the others, mostly because of the minimal toxic chemical use and reduced toxic waste emission^7,8^. Therefore, biological synthesis is preferable in terms of environmental protection. Unfortunately, AgNPs in general are known to impact some ecological processes, for example primary productivity, decomposition or nitrogen cycling. The presence of AgNPs in the soil can lead to the decrease of the quantity and activity of soil microbiota. AgNPs are also proved to be able to accumulate in plant tissues^9,10^. So, despite the green synthesis of AgNPs being an eco-friendly method, bio-manufactured nanoparticles can still pose a threat to the natural environment and their potential ecotoxic effect remains unknown^11^. In the case of biogenic AgNPs, the Among the organisms used in the biological method of AgNPs synthesis, filamentous fungi seem to be the most advantageous, as they are easy to cultivate, grow fast and secrete high amounts of extracellular compounds, serving as reducing and stabilizing agents. Moreover, filamentous fungi show high metal tolerance and the ability of metal bioaccumulation^12–14^. Various fungal species, such as *Fusarium*,* Aspergillus*,* Trichoderma*,* Cladosporium*,* Alternaria*,* Phytophora*,* Metarhizium*,* Beauveria*,* Isaria*,* Trametes*,* Phanerochaete*,* Ganoderma* and *Gloeophyllum* have been proven to successfully produce AgNPs with antimicrobial properties^13,15–21^. Wood decay fungi are especially worthy of extended attention in the terms of AgNPs synthesis due to their enzymatic abilities. Unlike white rot fungi, the information about brown rot fungal species used in AgNPs production are scarce. However, it was reported that *Gloeophyllum striatum* can perform the silver nitrate reduction^19^. Therefore, *G. striatum* DSM 9592 was chosen to perform the AgNPs synthesis.

In our previous study, the AgNPs production using *G. striatum* DSM 9592 was conducted in four different synthesis conditions – at 28 ºC with shaking, 28 ºC without shaking, 4 ºC with shaking and 4 ºC without shaking. Then, the antimicrobial potential of obtained nanoparticles was evaluated. It was found that all AgNPs were active against Gram-positive and Gram-negative bacterial strains and their potential varied depending on the synthesis scheme. Although no clear tendency of one synthesis scheme to be better than others was observed, it was noted that AgNPs synthesized at 4 ºC without shaking showed the best efficacy towards *Pseudomonas aeruginosa* reaching the lowest from all established minimal inhibitory concentration (MIC) values^22^. Proven antimicrobial potential of obtained AgNPs and the green method of their synthesis make *G. striatum*-derived AgNPs good candidates to be used on the industrial scale. Therefore, there is an urgent need for further examination of their potential adverse effects on the natural environment.

This paper presents research aiming in a comprehensive investigation of the ecotoxicity of mycogenic AgNPs. The study focused on the assessment of AgNPs toxicity towards organisms from different ecosystems and different trophic levels, shedding a light on their potentially negative impact in a broad ecological context. The tested organisms included soil bacteria *Pseudomonas putida* and *Pseudomonas moorei*, soil fungi *Trichoderma virens* and *Trichoderma reesei*, water bacteria *Aliivibrio fischeri*, water crustaceans *Artemia franciscana* and *Daphnia magna* and plants: *Spirodela polyrhiza*, *Sorgho saccharatum*, *Lepidium sativum* and *Sinapis alba*. All the tested species are representative for their environments and the changes of their viability can be indicative for adverse effect of xenobiotics. Some of them are used in the evaluation of acute toxicity as a standard organisms. Therefore, the use of mentioned species in the evaluation of AgNPs ecotoxic potential is highly relevant. To our best knowledge, such broad evaluation of ecotoxicity including organisms from different ecosystems and trophic levels regarding AgNPs originated from brown root fungi was performed for the first time. Moreover, our research shed a light on changes in AgNPs toxic effect depending on the synthesis conditions.

## Results

### The assessment of AgNPs activity towards soil microorganisms

**Fig. 1:**
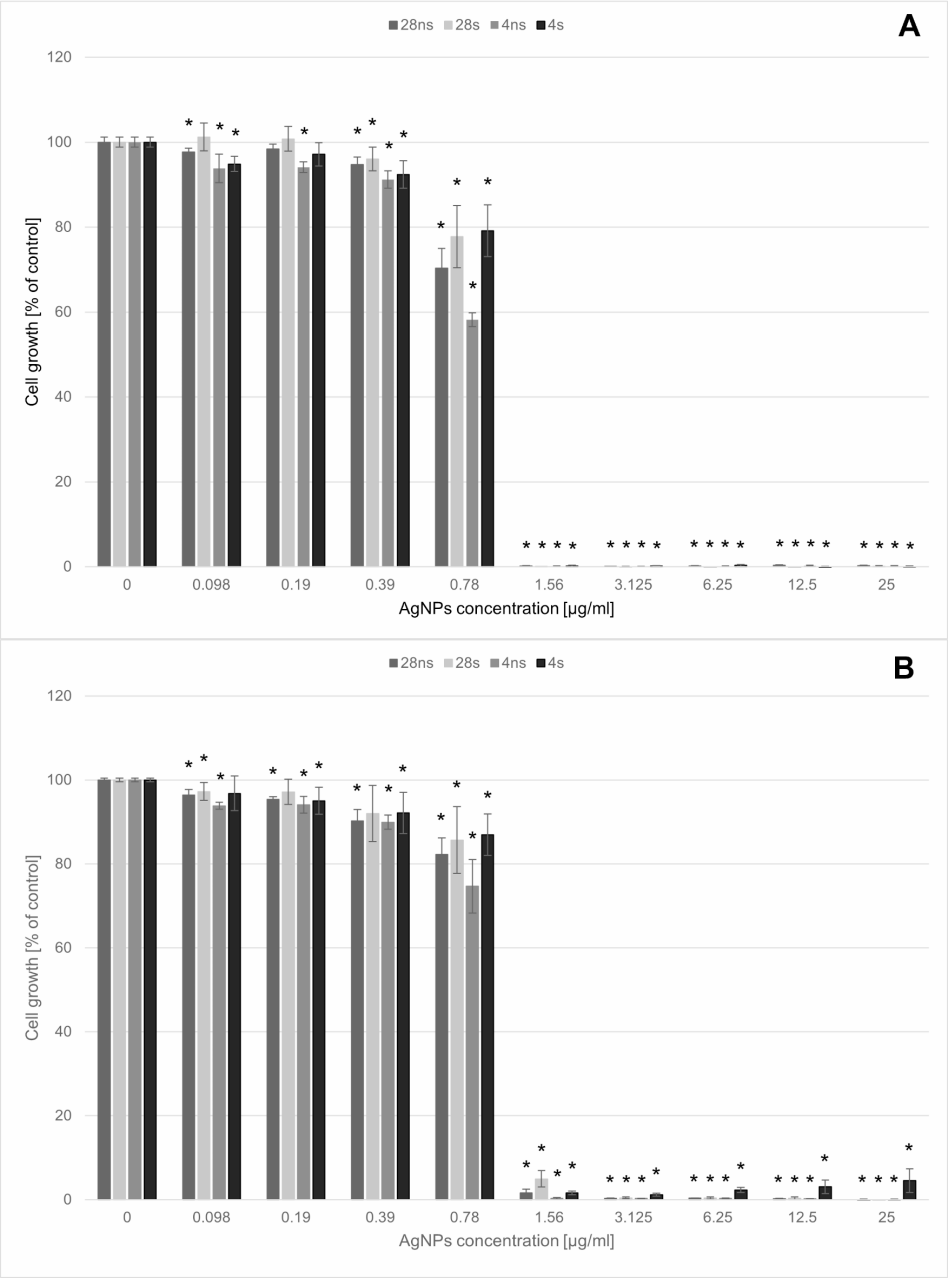
The mycogenic AgNPs activity against soil bacteria strains. (**A**) *Pseudomonas moorei* DSM 12,647, (**B**) *Pseudomonas putida* DSM 291. The results are shown as average percent values with standard deviations of optical density (OD) of the biotic control. The statistical significance was estimated using a one-way analysis of variance (ANOVA) test with * *p* < 0.05 and is shown by an asterisk.

**Fig. 2:**
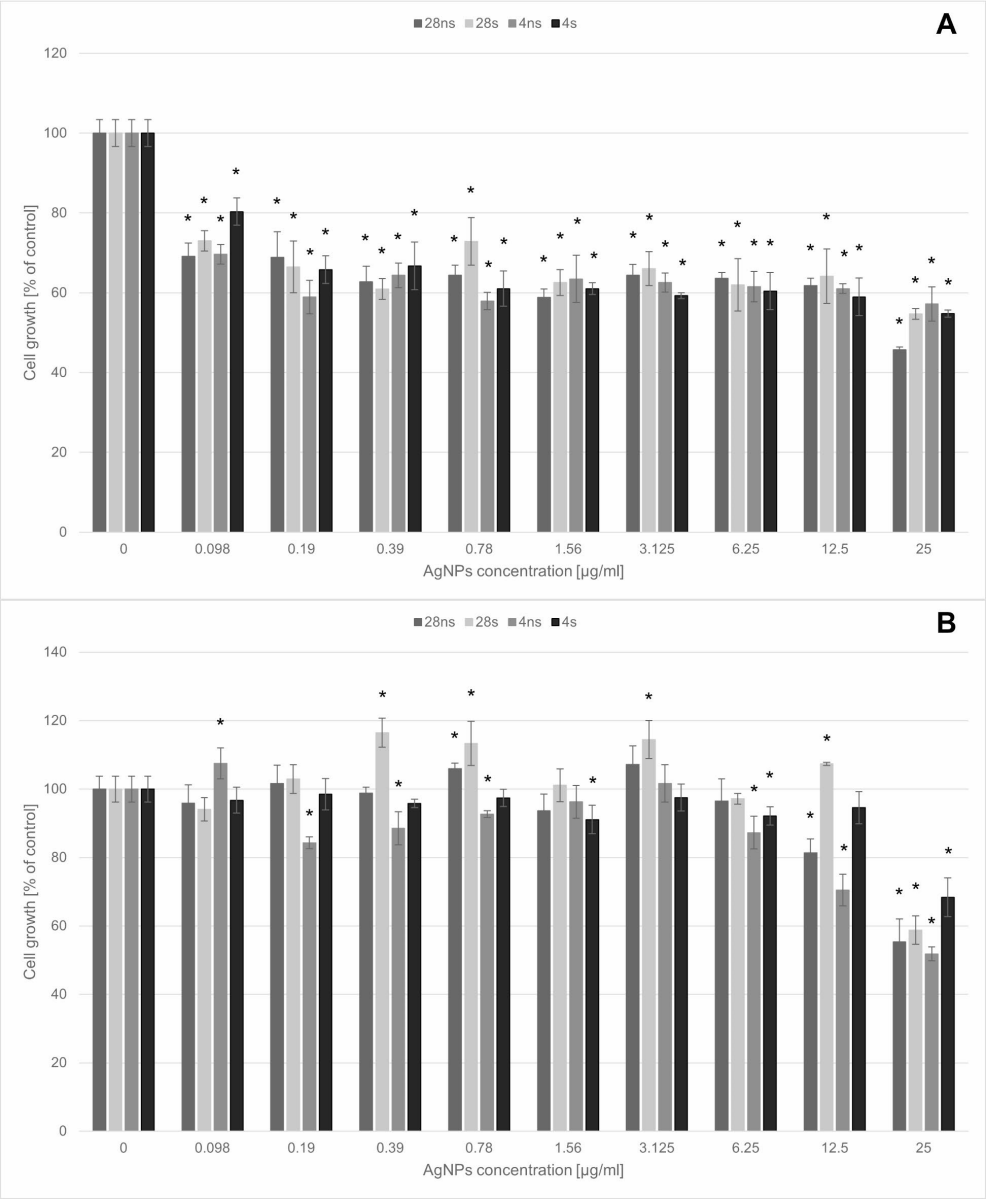
The mycogenic AgNPs activity against soil fungal strains. (**A**) *Trichoderma reesi* QM 9414, (**B**) *Trichoderma virens* DSM 1963. The results are shown as average percent values with standard deviations of optical density (OD) of the biotic control. The statistical significance was estimated using a one-way analysis of variance (ANOVA) test with * *p* < 0.05 and is shown by an asterisk.

The evaluation of the *G. striatum*-derived AgNPs activity against soil bacterial (Fig. 1) and fungal (Fig. 2) strains proved that bacterial strains were more susceptible to AgNPs action than fungal strains. The minimal inhibitory concentration (MIC_95_) for both tested bacterial strains reached 1.56 µg/mL. However, it was shown that *P. moorei* was more sensitive to 4ns AgNPs action at the concentration 0.78 µg/mL compared to the other bacterial strain tested.

Soil fungi occurred to be more resistant to AgNPs activity and the MIC values were higher than the tested concentration range. However, slight alternations of fungal growth in the presence of AgNPs were noticed. In the case of *T. virens*, all tested AgNPs caused up to 30% growth inhibition in concentrations 0.098–12.5 µg/mL. In the concentration of 25 µg/mL, the growth inhibition caused by 28ns AgNPs was even higher, reaching about 50%. *T. virens* was more tolerant to the presence of AgNPs in the growth environment. Only 28ns AgNPs and 4ns AgNPs had a negative influence causing about 20% growth inhibition in the concentration of 12.5 µg/mL. 50% growth inhibition was caused by 25 µg/mL 4ns AgNPs.

### AgNPs toxicity towards water organisms

#### *Aliivibrio fischeri*

The toxic effect of AgNPs on *A. fischeri* (Table 1) was established based on the changes in the bioluminescence intensity. It was proven that after 30 min of exposition, all types of AgNPs were active towards the tested bacterial strain with the EC_50_ values of 8.191, 7.855, 7.052 and 1.096 µg/mL for 28ns AgNPs, 28s AgNPs, 4ns AgNPs and 4s AgNPs, respectively. Both nanoparticle types synthesized at 4 ºC showed a stronger inhibitory effect on bioluminescence than the other analyzed AgNPs. It occurred that 28ns AgNPs were the least active against *A. fischeri* after the full time of incubation. The most severe effect on *A. fischeri* was caused by 4s AgNPs.

**Table 1:** The toxic effect of AgNPs on water bacterium *Aliivibrio fischeri* DSM 7151.

	28ns	28s	4ns	4s
0 min	15.093	11.584	15.801	12.057
5 min	11.638	9.544	11.191	9.507
15 min	9.326	8.721	8.105	7.709
30 min	8.191	7.855	7.052	7.096

The values shown in the table represent EC_50_ parameter corresponding to the concentration of AgNPs [µg/mL] causing the 50% inhibition of bioluminescence at 0, 5, 15 and 30 min of incubation.

#### Water crustaceans

**Fig. 3:**
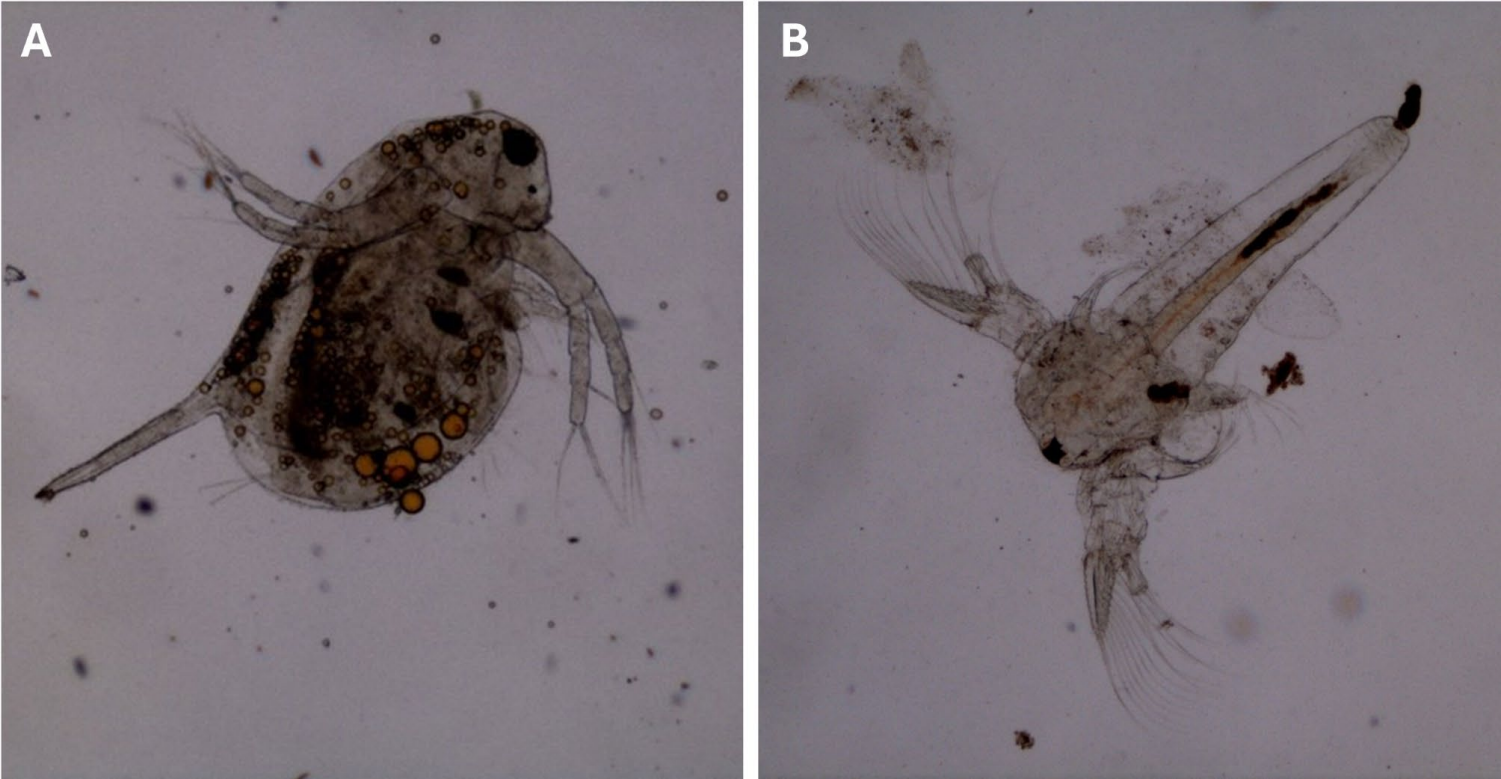
Microscopic image (400x magnification) of tested crustaceans. (**A**) *Daphnia magna* after 48 h exposition to 12.5 µg/mL AgNPs synthesized in 4 ºC without shaking and (**B**) *Artemia franciscana* after 48 h exposition to 25 µg/mL AgNPs synthesized in 4 ºC without shaking.

The ecotoxicity of mycogenic AgNPs towards water crustaceans (Table 2) was established with the use of freshwater *D. magna* and saline water *A. franciscana* (Fig. 3). The obtained results showed that *D. magna* was more susceptible to the presence of all tested AgNPs types in the growth environment compared to *A. franciscana*. After 24 h of incubation, the EC_50_ values established for *D. magna* were 0.029, 0.028, 0.042 and 0.029 µg/mL for 28ns AgNPs, 28s AgNPs, 4ns AgNPs and 4s AgNPs, respectively. After 48 h of incubation, the EC_50_ were 0.026, 0.027, 0.027 and 0.026 µg/mL in the order above. There were no significant differences in the toxic effect of AgNPs tested on *D. magna*. In *A. franciscana* the EC_50_ values were higher: 67.112, 58.440, 56.584, 61.736 µg/mL after 24 h of incubation and 26.162, 10.965, 23.945, 19.867 µg/mL after 48 h of incubation for 28ns AgNPs, 28s AgNPs, 4ns AgNPs and 4s AgNPs, respectively. Here, the tendency of AgNPs synthesized with shaking to be more toxic towards *A. franciscana* was observed and the most severe effect was caused by 28s AgNPs.

**Table 2:** The toxic effect of AgNPs towards water crustaceans *Daphnia magna* and *Artemia franciscana*.

		28ns	28s	4ns	4s
24 h	*Daphnia magna*	0.029	0.028	0.042	0.029
	*Artemia franciscana*	67.112	58.440	56.584	61.736
48 h	*Daphnia magna*	0.026	0.027	0.027	0.026
	*Artemia franciscana*	26.162	10.965	23.945	19.867

The values shown in the table represent EC_50_ parameter corresponding to the concentration of AgNPs [µg/mL] causing immobilization in 50% of tested individuals after 24 h and 48 h of incubation.

### AgNPs toxicity towards plants

#### *Spirodela polyrhiza*

**Fig. 4:**
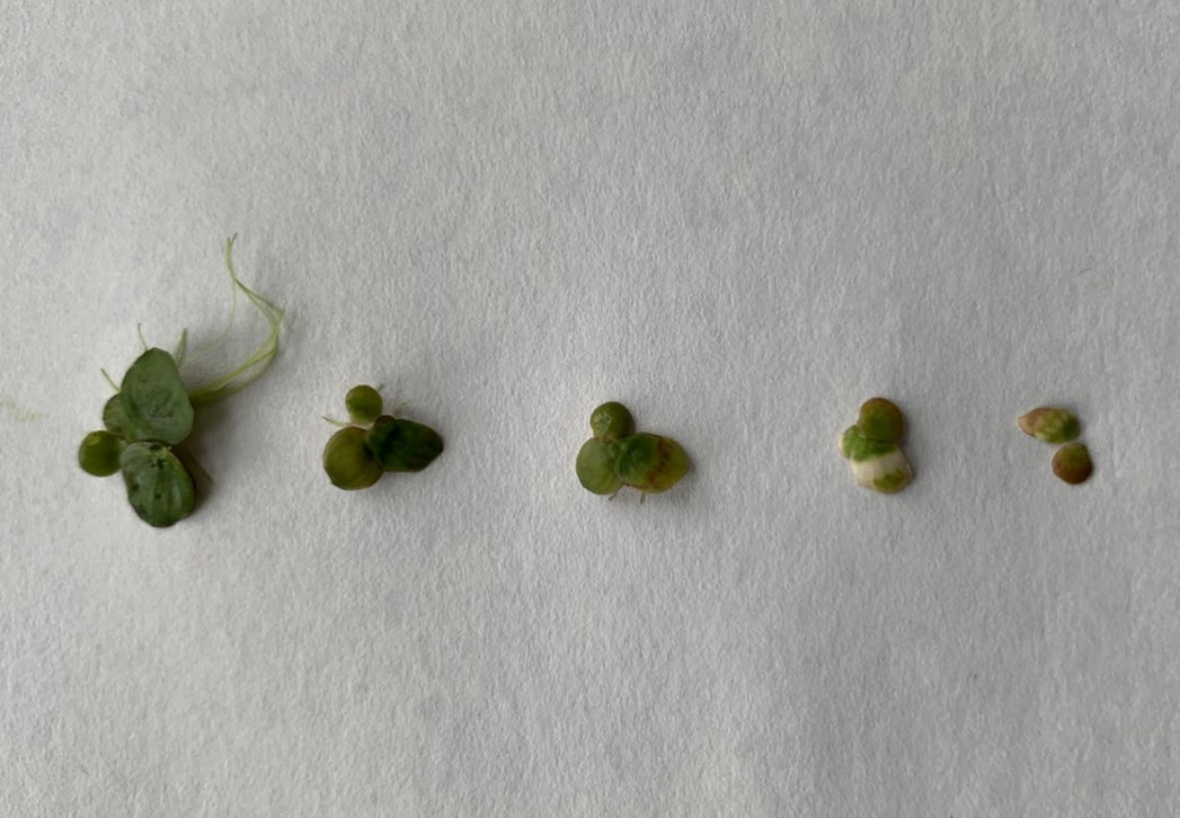
The effect of AgNPs synthesized in 28 ºC without shaking on *Spirodela polyrhiza* after 72 h of incubation. The AgNPs concentration was in the order from the left: 0, 0.78, 3.125, 12.5, 50 µg/mL.

The evaluation of the toxicity of. *G. striatum*-derived AgNPs towards *S. polyrhiza* (Fig. 4) indicated that all types of AgNPs affected the growth of the plant. After 72 h of incubation the EC_50_ values (Table 3) established for 28ns, 28s, 4ns and 4s AgNPs were 1.329, 2.819, 0.671 and 2.736 µg/mL, respectively. The obtained results indicate that the AgNPs synthesis scheme affected their toxicity potential – the effect of AgNPs synthesized without shaking was the most severe, and the 4ns AgNPs was the most toxic among tested nanoparticle types.

**Table 3:** The toxic effect of AgNPs towards tested plants *Spirodela polyrhiza*, *Sorgho saccharatum*, *Lepidium sativum* and *Sinapis alba*.

	28ns	28s	4ns	4s
*Spirodela polyrhiza*	1.329	2.819	0.671	2.736
*Sorgho saccharatum*	> 100	> 100	> 100	> 100
*Lepidium sativum*	> 100	> 100	> 100	> 100
*Sinapis alba*	> 100	> 100	> 100	> 100

The values shown in the table represent EC_50_ parameter corresponding to the concentration of AgNPs [µg/mL] causing 50% of plant growth inhibition after 72 h of incubation. The inhibitory effect on plant growth was determined based on changes in the first frond surface in *S. polyrhiza* or changes in the root lengths in *S. saccharatum*, *L. sativum* and *S. alba*.

#### *Sorgho saccharatum*, *Lepidium sativum*, *Sinapis alba*

The results of the phytotoxic effect investigation showed that crop plants used in the study were the least affected by the presence of mycogenic AgNPs among all organisms tested. It was established that for all tested plant species the EC_50_ values (Table 3) were above 100 µg/mL, which exceeded the tested concentration range of AgNPs.

## Discussion

As it was stated earlier, the water and soil ecosystems are the most endangered by the AgNPs presence due to their release to the environment. The estimated concentrations of AgNPs in soil are in the range of 0.24–729.23 ng/kg AgNPs. It is predicted that these numbers will have grown up to 10 µg/kg by 2050^23^. The concentration of dissolved Ag in natural water ecosystems varies from 0.03 to 500 mg/L. AgNPs are being detected in surface waters in different amounts ranging between 0.3 and 320 ng/L and higher in European countries^24^. These numbers show that organisms inhabiting soil and water ecosystems are in fact endangered by the AgNPs presence.

The first part of this study involved evaluating the effects of the presence of mycogenic AgNPs on soil bacteria and fungi. Our results showed that soil bacterial strains *P. putida* and *P. moorei* were more susceptible to the action of all tested AgNPs types than the two tested fungal strains. This phenomenon is in accordance with available data^23^. The MIC_95_ values established for both tested bacterial strains were 1.56 µg/mL for all AgNPs types. High susceptibility of *P. putida* to AgNPs both of biological and physical origin has been described in literature. Khan et al.^25^ revealed that AgNPs synthesized via the physical dispersion method showed antibacterial effect on the level higher than 80% in the concentration of 0.1 µg/mL. Gupta et al.^26^ investigated the effects of mycogenic AgNPs and proved that *P. putida* KT2440 was susceptible to their presence in the concentration of 0.4–0.8 µg/mL. In comparison, *G. striatum*-derived AgNPs synthesized in our study were less toxic. The adverse effects of AgNPs on whole microbial communities was also proven by Zhang et al.^27^ They found that the microbial community richness was decreased in the presence of AgNPs in the cucumber-planted soil. However, the positive influence of the biogenic AgNPs presence on microbial community abundance in soil was also proven^28^. That shows the necessity of further investigations to fully understand the impact of AgNPs on soil bacterial communities. Our results revealed that fungal strains *T. virens* and *T. reesei* showed lower susceptibility to the AgNPs presence in the environment compared to the bacterial strains tested. The high tolerance of *Trichoderma* species to the AgNPs presence has been described in literature. Oktarina et al.^29^ revealed that *T. virens* was able to grow in the AgNPs concentration of 300 mg/L and still reach about 80% of the biotic control growth level. In comparison, El-Ghany et al.^30^ investigated the impact of AgNPs synthesized by silver-resistant actinomycetes on mycotoxigenic fungi. Their research showed that biogenic AgNPs originating from two actinomycetes species inhibited the growth of *Aspergillus flavus* and *Aspergillus ochraceus* in the concentrations between 29.15 and 37.35 µg/mL. Moreover, it was shown that the presence of those AgNPs in the concentrations between 4.59 and 7.74 µg/mL inhibited the mycotoxin production completely. It indicates that the reaction to the AgNPs presence in the growth environment depends on the fungal species. In natural environments rhizosphere is colonized by multiple and diverse microbial communities. Bacteria can attach into the fungal surfaces forming fungal-bacterial biofilms that change the conditions in rhizosphere improving plant growth. Biofilm formation is one of the bacterial mechanisms of resistance to antimicrobial factors, including AgNPs. Consequently, it can be concluded that co-habiting the rhizosphere by both fungi and bacteria can indirectly enhance the protection of bacterial cells from AgNPs adverse effects^31,32^.

The second part of the present study involved investigating the impact of biogenic AgNPs on aquatic organisms. Water ecosystems are at risk of increased exposition to AgNPs not only due to the industrial wastewater efflux that may contain AgNPs from manufacturing-connected processes, but also because of AgNPs leaching from available consumer products^33^. Therefore, research on the possible negative effects of the presence of biogenic AgNPs in aquatic environment is necessary.

The investigation of the changes in bioluminescence intensity revealed that the aquatic bacterium *A. fischeri* was sensitive to the presence of tested AgNPs types. The EC_50_ values established after 30 min of exposition to AgNPs fluctuated around 7–8 µg/mL. In comparison, Binaeian et al.^34^ found that AgNPs synthesized by *E. coli* were more harmful towards *Vibrio fischeri* PTCC 1693 with the EC_50_ value after 30 min of exposition equal 34,550 ± 5,670 ppm. Interestingly, the research proved that the effect of biologically synthesized AgNPs was more severe compared to the effect of AgNPs of chemical origin. Different results were obtained by Gagné^35^, who investigated the effect of commercially available AgNPs on *V. fischeri* and estimated that the EC_20_ value after 30 min was above 500 µg/mL. Kyzioł-Komosińska et al.^36^ attempted at evaluating the toxicity of AgNPs released from textiles. They found that *V. fischeri* was less sensitive to the AgNPs leaching from the examined textiles than *D. magna* with EC_50_ values up to 46 times higher.

Our results concerning the toxicity of mycogenic AgNPs towards water crustaceans revealed that freshwater *D. magna* was more affected by the presence of all AgNPs types than saline *A. franciscana.* These results are in accordance with the findings of Zawadzka et al.^19^ investigating the effect of AgNPs derived from *G. striatum* DSM 10,335. High susceptibility of *D. magna* to the AgNPs influence was also confirmed by Ivask et al.^37^ In this research the size-dependent effect of AgNPs was established and calculated EC_50_ values ranged from 0.001 ± 0.014 mg Ag/L to 0.218 ± 0 mg Ag/L for 10 nm and 80 nm AgNPs, respectively. In contrast, Parkashi et al.^38^ proved that AgNPs in the concentration of 0.5 µg/l caused about 30% mortality in *D. magna* after 5 days of exposition. Similarly, a less severe effect of AgNPs on *D. magna* was observed by Aksakal et al.^39^, who compared the action of AgNPs of chemical and biological origin towards freshwater crustaceans. It was found that 48 h exposition to 50 ppb of chemical and biological AgNPs caused 19.3% and 31.8% mortality, respectively. These results highlight the possibility of biogenic AgNPs being more dangerous due to the penetration ability enhanced by the presence of biological compounds. Lower susceptibility of crustaceans from *Artemia* species was also described in the literature. Lish et al.^40^ found that after 48 h exposition of *A. salina* to commercially available AgNPs, the EC_50_ value was 50 mg/L. Bhakya et al.^41^ evaluated the effect of biogenic AgNPs on *A. salina*. It was revealed that the adverse effect of AgNPs was dose dependent, and after 48 h of exposition 50% of mortality was observed only at 150 and 200 µg/mL concentrations. It was noted, however, that after prolonging the exposition time, mortality was increasing. These results suggest that the time of exposition may be one of the important factors to be considered in ecotoxicological research concerning AgNPs. In another research study, the susceptibility of *A. salina* to AgNPs was notably higher, as the EC_50_ value established after 72 h was 10.70 ± 1.3 mg/L^42^. In the case of *Artemia* sp. the toxicity of AgNPs can be connected with their interaction with the cuticle of crustacean and modifying chitin structure by silver ions. Moreover, aggregates of AgNPs can be found in the crustaceans’ gut causing interferences in food uptake and leading to the death of starvation^43,44^. In daphnids, the bioaccumulation of AgNPs inside the organisms is caused mainly by ingestion, and nanoparticles can be found agglomerated in the gut. Moreover, AgNPs can accumulate under the carapace, in the brood chamber and can be attached to the external parts of the body^45,46^. As mechanisms of action of AgNPs on tested crustacean species do not differ significantly, the most convincing explanation for *Artemia* sp. being more resistant to AgNPs action is differences in external parameters of organisms’ environment. It was shown that increasing water salinity significantly reduces mortality of *Oncorhynchus mykiss* fry exposed to colloidal AgNPs^47^. The same phenomenon was also proven in the case of invertebrate *Saccostrea cucullata*, where the decrease of water salinity intensified the toxic effect of AgNPs on tested species^48^. This effect can be attributed to the reduced ion release from the surface of nanoparticles at higher water salinity^49^. Given above examples it can be concluded that the level of water salinity is the factor differentiating the toxic effect of mycogenic AgNPs on tested crustaceans.

The last part of the present research was the investigation of the AgNPs impact on plants. Here, the water plant *S. polyrhiza* and crop plants *S. saccharatum*, *L. sativum* and *S. alba* were taken into consideration. This kind of research is of particular value, because plants as primary producers may play a role of a transport pathway for AgNPs to the organisms of higher trophic levels^50^.

Our results showed that the growth of *S. polyrhiza* was inhibited by all tested AgNPs in a concentration-dependent manner. The established EC_50_ values varied between 0.671 and 2.819 µg/mL. The same tendency was proven by Radić et al.^6^, who investigated the effect of commercially available AgNPs on different species of duckweed, *Lemma minor*. It was observed that 0.5 and 1 mg/L AgNPs caused *L. minor* growth inhibition between 32 and 37% and 52–59%, respectively. Jang et al.^51^ found that gum-arabic coated AgNPs also caused growth inhibition of *S. polyrhiza* in a concentration-dependent manner. The tested AgNPs had a less severe effect on *S. polyrhiza* than mycogenic AgNPs in our research with the EC_50_ value of 13.39 ± 1.06 mg Ag/L. A less serious influence of chemical AgNPs on duckweed species *Landolita punctata* was also proven by Lalau et al.^4^, who found the EC_50_ value of tested AgNPs to be 6.84 ppm.

According to our research, the effect of mycogenic AgNPs on plant growth was the least severe compared to the other performed tests. The EC_50_ values for three tested plant species were above the tested concentration range, and the effect of AgNPs on the plant growth was not visible. The benign influence of chemical AgNPs on sorghum and white mustard growth was confirmed by Matras et al.^50^ They found that three different types of chemically synthesized AgNPs did not cause significant inhibitions either in root growth or seeds germination compared to the controls. Tomaszewska-Sowa et al.^52^ observed that *S. alba* roots growth was even stimulated by the presence of AgNPs. However, AgNPs showed adverse effects inhibiting the growth of *S. alba* hypocotyls in the highest concentration tested. The influence of AgNPs on plants is thought to be both species- and dose-dependent, as a different amount of AgNPs can promote or inhibit plant growth. It has been proven, however, that AgNPs inhibit root growth of other plant species, e.g. *Arabidopsis thaliana* roots growth was inhibited in the presence of 3 mg/L AgNPs^52–54^. It is worth noticing that AgNPs present in the aquatic or soil environment can have indirect effects on plant growth. As it was stated earlier, AgNPs can accidentally enter the water and terrestrial environments^2^. In the soil ecosystems, increased presence of AgNPs can lead to lowering the quality of the soil and negatively affect growth and abundance of microbial communities, included microorganisms supporting plant growth and nutrient cycling^27,55^. These alternations can influence plants development. Moreover, AgNPs in soil can themselves enter the plant organisms via roots. After crossing cellular covers, AgNPs can be transported symplasticaly or apoplasticaly to the inner parts of the plant and then accumulate for example in root meristem or epidermis. AgNPs can have adverse effects on seeds germination and growth of the roots. They can negatively influence processes that are crucial for the plant development, such as gaseous exchange, photosynthesis or transpiration rate. On the molecular level, the presence of AgNPs leads to enhanced reactive oxygen species production in plant cells. This can end in processes with potentially lethal outcomes, for example oxidation of the proteins, lipid peroxidation, changing the properties of cell membranes, impairing the activity of the enzymes, destroying nucleic acids or initiating programmed cell death^56^. As the phytotoxicity of AgNPs is mainly connected with oxidative stress, some antioxidant defense mechanisms can be developed in plant cells. These mechanisms involve the activity of enzymes of antioxidative properties, for example superoxide dismutase, catalase or glutathione reductase. Moreover, non-enzymatic antioxidants like ascorbate, glutathione or ascorbic acid and carotenoids can play a role in the protection of the cell against adverse AgNPs effects^57^. It can be concluded, that activated antioxidant defence mechanisms were the reason for the lack of sensitivity of tested crop plants to AgNPs action. Although studies of AgNPs impact on plants mostly suggest their adverse effects, a few confirmations of nanoparticles stimulatory influence can be found. Plant response to AgNPs can strictly depend on their dosage causing enhancement or inhibition of plant growth. While exposing on low or high concentrations of AgNPs has negative effects, exposure to specific, proper concentrations can improve plant growth, seed germination, chlorophyll content and fertilizer or water efficiency in plants^53,56^.

The ecotoxic effect of AgNPs may be altered depending on the stability of nanoparticles, as it constitutes one of the main factors determining their activity. Some environmental factors, such as pH, temperature, light exposure, presence of reactive species, dissolved oxygen content, dissolved organic matter content or presence of cations and ions can all affect the AgNPs stability, which can lead to changes in the behavior of nanoparticles as a xenobiotic in different ecosystems^19,58–60^. Therefore, it should be highlighted that further investigation of AgNPs ecotoxicological potential is highly needed and should include environmental matrices for better understanding the fate of nanoparticles and changes in their activity.

## Conclusion

In our previous study we described the synthesis of AgNPs by brown root fungus *G. striatum* DSM 9592 for the first time. It was proven that obtained nanoparticles possessed antibacterial activity that can vary depending on the synthesis scheme. This research provided a broad evaluation of the ecotoxicity potential of these mycogenic AgNPs towards organisms from different ecosystems and trophic levels. Among these organisms were soil fungi species, which made the analysis of AgNPs impact on soil microbiota more complete. It was proved that AgNPs can pose a threat to the organisms living in water and soil environments, but the sensitivity to their presence varied between the tested species. *D. magna* was the most affected among all tested organisms – here, the EC_50_ values for all AgNPs types tested were below 0.1 µg/mL. *S. saccharatum*, *L. sativum*, *S. alba* were the least sensitive to the presence of all AgNPs showing no adverse effects in the concentration of 100 µg/mL. It was also noted that the ecotoxic potential of biogenic AgNPs could vary depending on the synthesis scheme, e.g. in the case of *S. polyrhiza*. Our results confirmed the necessity of conducting ecotoxicological research regarding potential adverse effects of AgNPs on endangered ecosystems. In the era of the widespread use of AgNPs and ongoing search for new methods of their synthesis, the ecological safety of their utilization should become one of the most fundamental priorities. The future directions of this kind of research should cover the exact mechanisms of AgNPs impact on different organisms and evaluating how different environmental factors such as salinity, pH, temperature, as well as AgNPs properties like stability can affect the toxicological effect of nanoparticles.

## Methods

### Materials

All toxkits used in the presented study, namely: Daphtoxkit F magna (MicroBioTests Inc.) for the evaluation of AgNPs toxicity towards *D. magna*, Artroxkit M (MicroBioTests Inc.) for the determination of AgNPs toxic effect towards *A. franciscana*, Duckweed toxkit F (MicroBioTests Inc.) for the investigation of toxicological impact of AgNPs on *S. polyrhiza* and Phytotoxkit (MicroBioTests Inc.) for evaluating the toxic potential of AgNPs towards crop plants were obtained from Tigret Sp. z o.o. (Poland). During conducting the experiments with the use of toxktis, the following organism species were used: *D. magna*, *A. franciscana*, *S. polyrhiza*, *S. saccharatum*, *L. sativum*, *S. alba*. All mentioned species are commonly used in the evaluation of the acute toxicity and are prescribed in the manufacturer’s protocols. The *A. franciscana* cysts, *D. magna* ephippia, *S. polyrhiza* turions and *S. saccharatum*, *L. sativum*, *S. alba* seeds came as a part of the purchased toxkits (Tigret Sp. z o.o., Poland). The tested strains *P. putida* DSM 291, *P. moorei* DSM 12,647, *A. fischeri* DSM 7151 and *T. virens* DSM 1963 were acquired from the German Collection of Microorganisms and Cell Cultures GmbH (Germany) as well as fungal strain *G. striatum* DSM 9592 used for AgNPs synthesis. The fungal strain *T. reesei* QM 9414 was obtained from the American Type Culture Collection (ATCC). *P. putida*, *P. moorei*, *T. virens* and *T. reesei* are the ubiquitously present microorganism in the soil environment, therefore choosing these strains for the evaluation of AgNPs impact on soil microbiota seemed appropriate. *A. fischeri* is commonly used as a standard organism in the evaluation of the toxicity towards aquatic ecosystems. Silver nitrate (Sigma-Aldrich) was purchased from Merck (Poland). Sabouraud dextrose broth (Difco) and Mueller-Hinton broth (BBL) were bought from Becton Dickinson (Poland).

### Silver nanoparticles synthesis

Silver nanoparticles were synthesized according to the previously described method^22^. The process was conducted with the use of *G. striatum* DSM 9592 supernatant and AgNO_3_ as a nanoparticle precursor. *G. stratium* DSM 9592 was cultivated in the Sabouraud dextrose broth supplemented with 2% glucose in 28 ºC on a rotary shaker at 120 rpm for 120 h. After incubation period, fungal biomass was filtered through sterile filter paper and suspended in sterile deionized water. Prepared sample was again incubated in the same conditions for 120 h. Then, the biomass was filtered to obtain supernatant. The prepared fungal filtrate was divided into four parts. Each part was supplemented with AgNO_3_ stock which reached the final concentration of 5 mM. Prepared samples was incubated in the dark at four different process conditions – at 28 ºC with shaking (28s AgNPs), 28 ºC without shaking (28ns AgNPs), 4 ºC with shaking (4s AgNPs) and 4 ºC without shaking (4ns AgNPs).

### AgNPs activity assessment against soil microorganisms

#### Soil bacteria

The antibacterial potential of fungi-derived AgNPs against *P. putida* DSM 291 and *P. moorei* DSM 12,647 was determined using the protocol described by Nowak-Lange et al.^61^ with modifications. Both tested strains were cultivated in Mueller-Hinton broth medium. The tested AgNPs concentrations were 0.098, 0.19, 0.39, 0.78, 1.56, 3.13, 6.25, 12.5 and 25 µg/mL and the dilutions were made in the appropriate medium. Biotic and abiotic controls were also prepared. The bacterial susceptibility to the investigated AgNPs was determined based on the optical density (OD) measurement at a wavelength of 630 nm carried out with a Multiskan™ FC Microplate Photometer (Thermo Fischer Scientific, Pudong, China). The minimal inhibitory concentration (MIC_95_) values were also determined as the lowest concentration of AgNPs that inhibited bacterial growth. MIC_95_ values were expressed in µg/mL.

#### Soil fungi

The antifungal potential of investigated AgNPs was determined by the microdilution method in accordance with the Clinical and Standard Laboratory Institute (CSLI) M38 (3rd Edition) guideline dedicated to filamentous fungi with some modifications. The evaluation was performed in 96-well cell culture plates. Inocula of both tested strains – *T. virens* DSM 1963 and *T. reesei* QM 9414 were prepared in Sabouraud dextrose broth and reached the final density of 8 × 10^6^ spores/mL. Dilutions of AgNPs were prepared in the same medium and reached the concentration range of 0.098–25 µg/mL. Biotic and abiotic controls were also prepared. After 48 h of incubation at 28 ºC the OD was measured in the same way as described in point 4.3.1. MIC_95_ values were also established.

### AgNPs ecotoxicity assessment against water organisms

#### *Aliivibrio fischeri*

Toxicity of AgNPs towards *A. fischeri* DSM 7151 was evaluated according to the ISO 11348-1:2007(E) standard. A single colony of *A. fischeri* was transferred into dedicated liquid medium and cultivated at 20 ºC/180 rpm for 22 h. After cultivation, the competent bacterial cells were diluted to the turbidance of 2500 FAU in the fresh protective medium prepared earlier according to the mentioned standard. Obtained bacterial suspension was divided into 100 µL stocks and stored at -80 ºC. Before the procedure, the competent bacterial cells stored in stocks were refrozen and diluted in dedicated medium chilled to 15 ºC. Next, 12 mL of medium was added to each 100 µL of bacterial suspension. The evaluation was performed in dedicated 96-well plates with black frames and white wells and the concentration range of the tested AgNPs was 0.098–25 µg/mL. 20% NaCl was used as a dilutant. Changes in bioluminescence intensity indicating changes in the bacterial cells viability in the presence of AgNPs were measured on a SpectraMax i3 Multimode Microplate Reader (Molecular Devices, USA) after 0, 5, 15 and 15 min of incubation. Between measurements, the tested plates were stored at 15 ºC in darkness. Half-maximal effective response (EC_50_) values for each measurement point were established as 50% inhibition of bioluminescence compared to the biotic control.

#### *Artemia franciscana*

The evaluation of AgNPs toxicity towards saline crustaceans *A. franciscana* was performed using the Arthroxkit M test, which was carried out in accordance with the producer’s protocol and ISO/TS 20787:2017 standard. The cysts of *A. franciscana* were incubated in saline water at 25 ºC and constant lightness of 3000 lx for 48 h. After hatching, the larvae were transferred to 24-well plates, where AgNPs dilution was prepared in the same medium reaching the concentration range of 6.25–100 µg/mL. The plates were incubated for 48 h at 25 ºC in darkness. After the incubation period, EC_50_ values were established based on the number of immobile *A. franciscana* individuals. The untreated larvae were used as a biotic control.

#### *Daphnia magna*

The assessment of AgNPs toxicity towards freshwater crustaceans *D. magna* was performed with the use of Daphtoxkit F magna according to the producer’s protocol and ISO 6341:2012 standard. The ephippia of *D. magna* were incubated in fresh water at 20 ºC and constant lightness of 6000 lx for 72 h. After incubation, the motile individuals were transferred to 6-well plates with AgNPs diluted in the same medium to the concentration range of 0.005–0.1 µg/mL. The plates were incubated for 48 h at 20 ºC in darkness. After incubation, EC_50_ values were determined based on the number of immobile larvae. The untreated individuals were used as a biotic control.

### AgNPs ecotoxicity assessment against plants

#### *Spirodela polyrhiza*

The investigation of AgNPs toxicity towards *S. polyrhiza* was conducted using the Duckweed toxkit F according to the producer’s protocol and ISO 20079:2005(E) standard. The turions of *S. polyrhiza* were incubated in the dedicated Stainberg medium at 25 ºC and constant lightness of 6000 lx for 72 h. After incubation, germinated turions were transferred to 48-well plates with diluted AgNPs in the same medium to the concentration range of 0.78–25 µg/mL. The plates were incubated for 72 h at 25 ºC and constant lightness of 6000 lx. EC_50_ values were established based on the changes in the first fonds surface size before and after incubation with AgNPs. Germinated turions cultivated in the medium without AgNPs were used as a biotic control. Differences in fonds size were determined using the ImageJ software (National Institutes of Health and the Laboratory for Optical and Computational Instrumentation, University of Wisconsin, USA).

#### *Sorgho saccharatum*, *Lepidium sativum*, *Sinapis alba*

The phytotoxic activity of mycogenic AgNPs was investigated using the Phytotoxkit according to the producer’s protocol. In this test, the effect of AgNPs in the concentrations of 50 and 100 µg/mL towards three plant species, namely *S. saccharatum*, *L. sativum* and *S. alba*, was checked. AgNPs diluted in sterile deionized water were transferred to the plates with a layer of sterile filter paper. Then, the seeds of each plant were soaked in sterilized deionized water and placed on the test plates. The prepared plates were closed with the transparent covers and incubated in the vertical position for 72 h at 25 ºC in darkness. After incubation, the root lengths were measured using the ImageJ software (National Institutes of Health and the Laboratory for Optical and Computational Instrumentation, University of Wisconsin, USA). EC_50_ values were established based on the differences in roots lengths between plants cultivated with and without AgNPs addition.

### Results analysis

#### Statistical analysis

Each experiment was conducted in four replicates (*n* = 4). The results of AgNPs activity against soil microorganisms were analyzed with the use of a one-way ANOVA test with * *p* < 0.05 to estimate the statistical significance. The estimation and all needed calculations were carried out by using Excel, Microsoft Office 2021 (Microsoft Corporation, Redmont, WA, USA). The results shown in the figures are expressed as the average values with the standard deviation (SD).

#### Half-maximal effective response (EC_50_) estimation

The EC_50_ values were established based on the calibration curves equations obtained by using Excel, Microsoft^®^ Office 2021 (Microsoft Corporation, Redmont, WA, USA) and meant the concentration of AgNPs causing the 50% of biological response in the tested organisms.

## Data Availability

The data that support the findings of this study are available from the corresponding author upon reasonable request.
